# Supplementary figures and images for: Global emergence of double and multi-carbapenemase producing organisms: epidemiology, clinical significance, and evolutionary benefits on antimicrobial resistance and virulence
Source: Microbiol Spectr. 2024 Jun 11;12(7):e00008-24. doi: 10.1128/spectrum.00008-24 (PMC11218513; doi:10.1128/spectrum.00008-24)

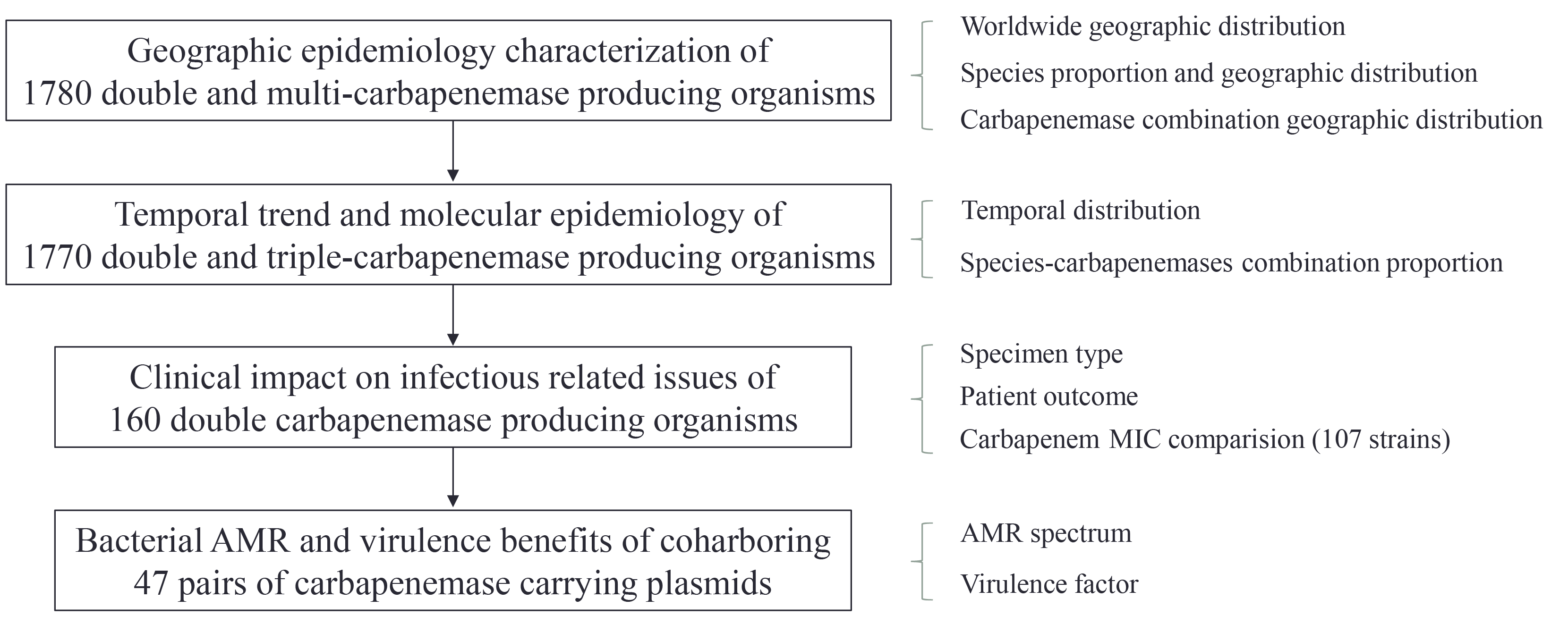

Supplement: Supplemental material — Fig. S1. [file spectrum.00008-24-s0001.tif]
